# Cholera Transmission in Ouest Department of Haiti: Dynamic Modeling and the Future of the Epidemic

**DOI:** 10.1371/journal.pntd.0004153

**Published:** 2015-10-21

**Authors:** Alexander Kirpich, Thomas A. Weppelmann, Yang Yang, Afsar Ali, J. Glenn Morris, Ira M. Longini

**Affiliations:** 1 Department of Biostatistics, College of Public Health and Health Professions and College of Medicine, University of Florida, Gainesville, Florida, United States of America; 2 Emerging Pathogens Institute, University of Florida, Gainesville, Florida, United States of America; 3 Department of Environmental and Global Health, College of Public Health and Health Professions, University of Florida, Gainesville, Florida, United States of America; 4 Department of Medicine, College of Medicine, University of Florida, Gainesville, Florida, United States of America; University of Minnesota, UNITED STATES

## Abstract

In the current study, a comprehensive, data driven, mathematical model for cholera transmission in Haiti is presented. Along with the inclusion of short cycle human-to-human transmission and long cycle human-to-environment and environment-to-human transmission, this novel dynamic model incorporates both the reported cholera incidence and remote sensing data from the Ouest Department of Haiti between 2010 to 2014. The model has separate compartments for infectious individuals that include different levels of infectivity to reflect the distribution of symptomatic and asymptomatic cases in the population. The environmental compartment, which serves as a source of exposure to toxigenic *V. cholerae*, is also modeled separately based on the biology of causative bacterium, the shedding of *V. cholerae* O1 by humans into the environment, as well as the effects of precipitation and water temperature on the concentration and survival of *V. cholerae* in aquatic reservoirs. Although the number of reported cholera cases has declined compared to the initial outbreak in 2010, the increase in the number of susceptible population members and the presence of toxigenic *V. cholerae* in the environment estimated by the model indicate that without further improvements to drinking water and sanitation infrastructures, intermittent cholera outbreaks are likely to continue in Haiti.

## Author Summary

Based on the model-fitted trend and the observed incidence, there is evidence that after an initial period of intense transmission, the cholera epidemic in Haiti stabilized during the third year of the outbreak and became endemic. The model estimates indicate that the proportion of the population susceptible to infection is increasing and that the presence of toxigenic *V. cholerae* in the environment remains a potential source of new infections. Given the lack of adequate improvements to drinking water and sanitation infrastructure, these conditions could facilitate ongoing, seasonal cholera epidemics in Haiti.

## Introduction

After a massive earthquake struck the island nation of Haiti in 2010, the introduction of an altered El Tor biotype of *Vibrio cholerae* O1 has led to one of the largest cholera outbreaks in recent history [1] [2] [3]. Almost four years after the identification of the first cholera cases, the transmission appears to have temporarily slowed, however the future of the cholera epidemic in Haiti remains uncertain [4]. After the initial isolation of toxigenic *V. cholerae* O1 from surface water monitoring sites in the Ouest Department of Haiti in 2012 and 2013, there is evidence that the frequency of isolation from the environment has actually increased between 2013 and 2014 [5] [6]. In the absence of ongoing transmission, the presence of toxigenic *V. cholerae* O1 in the aquatic environment has left the international scientific community divided on the possibility that the causative bacterium has established environmental reservoirs in the surface waters of Haiti [7] [8] [9]. If this were to be the case, the goal of cholera elimination from the island of Hispaniola by 2022 would be more challenging, with the potential for cholera to become endemic in Haiti [10].

To assist in the planning and allocation of resources necessary to mitigate the outbreak, mathematical models have been developed to investigate the underlying dynamics of cholera transmission in Haiti. [11]. However, despite empirical evidence that *V. cholerae* O1 is increasingly present in the surface water as reported cases continue to decline, none of the previous models have considered the role of environmental reservoirs in cholera transmission [6]. Though the environmental compartment has been included in the models, it is assumed that *V. cholerae* O1 occupy a transient state where after being shed from the human host they will eventually become removed from the environment at a constant rate of decay [12]. However, in endemic countries, this assumption is often likely to be false; where *V. cholerae* O1 is able to persist and multiply in the environment in response to an influx of nutrients into surface waters after rainfall events or increases in water temperature leading to recurrent outbreaks after interepidemic periods where very few cases were reported [13]. Since both water temperature and rainfall have been associated with increased isolation frequency of toxigenic *V. cholerae* O1 in Haiti [6], a dynamic cholera transmission model was created with the additional mechanism by which the environmental compartment responds to factors such as precipitation and surface water temperature that increase the concentration of the organism in the aquatic environment. Hopefully, these extra parameters will assist in the understanding of the underlying processes of cholera transmission in Haiti and allow for more accurate prediction of the potential for future outbreaks.

## Methods

To reflect the basic differences in the modes of transmission, the model incorporates both the short cycle transmission from human-to-human and long cycle transmission from human-to-environment and environment-to-human. The short route relies on data suggesting that toxigenic *V. cholerae* assumes a short-lived hyperinfectious state immediately after passage from the human intestine [14]. This facilitates rapid transmission of *V. cholerae* from one person to another, often related to personal hygiene practices within the household. Alternatively, transmission may occur when *V. cholerae* is acquired from contaminated drinking water or by contact with the aquatic environment. The presence of toxigenic *V. cholerae* in the aquatic environment may reflect contamination of water sources by feces from an infected individual, and/or the existence of an aquatic reservoir in which the microorganism can persist for months to years [13]. Transmission through this aquatic route, while still having the potential for being relatively rapid, tends to involve more time than the short cycle transmission between humans.

In the model, separate compartments for infectious symptomatic and infectious asymptomatic cases are used, even though it is not possible to estimate the size of the asymptomatic compartment. This is done to increase the model flexibility and to provide the option for sensitivity analysis. Dichotomization between symptomatic and asymptomatic cases also provides the option to address different infectivity levels for symptomatic and asymptomatic infections. The model has the following compartments:

- *S*(*t*)—number of susceptible people at time *t*.
- *A*(*t*)—number of asymptomatic people at time *t*.
- *I*(*t*)—number of symptomatic people at time *t*.
- *R*(*t*)—number of recovered people at time *t*.
- *W*(*t*)—bacteria concentration in the water at time *t* (environmental compartment.)

**Fig 1:**
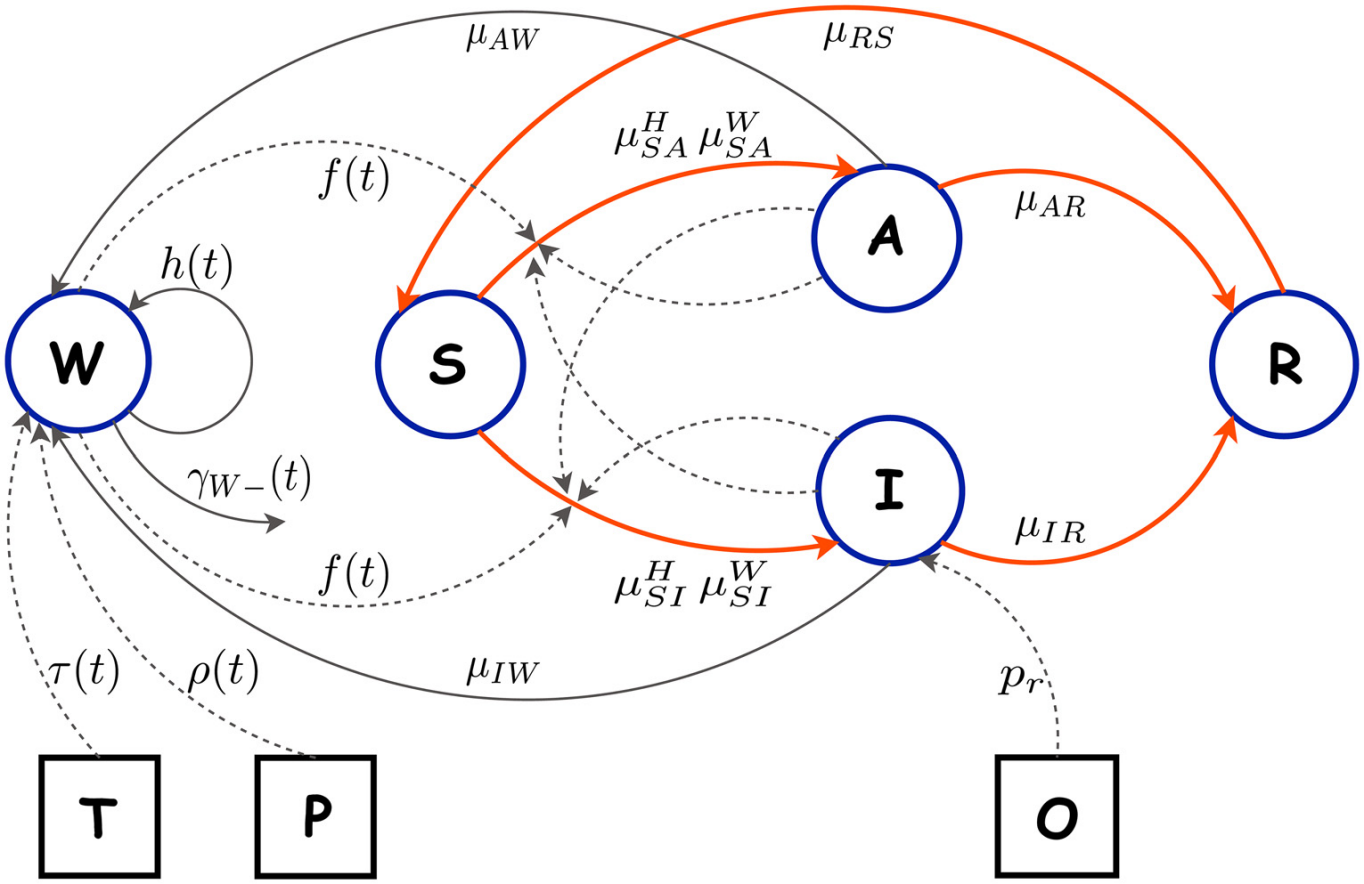
Compartmental model diagram. The unobserved compartmental *SIRS* model is linked to the observed data via a set of modeling assumptions. Blue boundary circular objects are the unobserved compartments of the *SIRS* model with the environmental compartment *W*. Black boundary square objects represent the collected observed data where *O* stands for the reported incidence and *T* and *P* stand for the environmental measurements of temperature and precipitation respectively. Temperature and precipitation affect the environmental reservoir *W*. The orange arrows indicate the movement of individuals between the human compartments. The grey arrows represent other processes in the model that do not directly involve the movement of humans between compartments. The actual bacterial movements such as human shedding and bacteria death are represented by solid grey lines. The other processes in the model such as the influence of temperature and precipitation on bacterial growth, the influence of aquatic reservoir and infected humans on transmission, and the relationship between the reported incidence *O* and the unobserved symptomatic incidence are represented by dashed grey lines. Please refer to S1 Text for more details on the model formulation, parametrization and relevant assumptions.

The model diagram and the relationships between the model compartments and the observed data are summarized visually in the diagram provided in Fig 1.

In the model the movement of people between the compartments *S*, *A*, *I*, *R* is considered along with the growth and death of bacteria within the environmental compartment *W*. The system of ordinary differential equations (ODE) that defines our model has the form:

$$\begin{array}{cc} \frac{dS(t)}{dt}= & \mu_{RS}R\left(t\right)-\left(\mu_{SA}^{W}+\mu_{SI}^{W}\right)S\left(t\right)f\left(t\right)-\left(\mu_{SA}^{H}+\mu_{SI}^{H}\right)S\left(t\right)(A(t)+I(t))\\ \frac{dA(t)}{dt}= & \mu_{SA}^{W}S\left(t\right)f\left(t\right)+\mu_{SA}^{H}S\left(t\right)(A(t)+I(t))-\mu_{AR}A\left(t\right)\\ \frac{dI(t)}{dt}= & \mu_{SI}^{W}S\left(t\right)f\left(t\right)+\mu_{SI}^{H}S\left(t\right)(A(t)+I(t))-\mu_{IR}I\left(t\right)\\ \frac{dR(t)}{dt}= & \mu_{AR}A\left(t\right)+\mu_{IR}I\left(t\right)-\mu_{RS}R\left(t\right)\\ \frac{dW(t)}{dt}= & g\left(t\right)(\mu_{AW}A\left(t\right)+\mu_{IW}I\left(t\right))+h\left(t\right)m\left(t\right)W\left(t\right)-\gamma_{W-}\left(t\right)W\left(t\right) \end{array}$$ (1)

In the model equations *μ* and *γ* indicate the transition rates with corresponding subscripts and superscripts that indicate the direction and the nature of the movement. The superscript *H* indicates the rates responsible for human-to-human transmission and superscript *W* indicates the rates responsible for environment-to-human transmission.

To address the dynamic of the environmental compartment three main process that affect bacterial growth and survival in the environment were considered.

The first process is the influx of bacteria via shedding by infected human hosts into the environment. Once shed into the environment the bacteria provide a source of exposure for susceptible humans. Those processes are modeled by the functions:

$$f\left(t\right)=\frac{W(t)}{\kappa +W(t)}\;\;\text{and}\;\;g\left(t\right)=\frac{\rho (t)}{\delta +\rho (t)}.$$

The notations *ρ*(*t*) for the total weekly precipitation in mm and *τ*(*t*) for average weekly temperature in degrees Celsius at time *t* are used. Here *κ* and *δ* are the threshold parameters.

The second process is the multiplication of the bacteria in the environment, which is affected by both temperature and precipitation. This process is modeled by functions *h*(*t*) and *m*(*t*):

$$h\left(t\right)=\alpha \exp[-\frac{{\left(\rho \left(t\right)-\rho_{c}\right)}^{2}}{2\sigma^{2}}]+\beta \tau \left(t\right)\;\;\text{and}\;\;m\left(t\right)=1-\frac{W(t)}{\chi }=\frac{\chi -W(t)}{\chi }.$$

Here *α*, *ρ*_*c*_, *σ* and *β* are the parameters of interest and *χ* is the cap designed to constrain the excessive growth of bacteria in the environment.

The functional form *m*(*t*) represents the logistic growth multiplier widely used in population dynamic models. This multiplier allows the growth to be proportional to the current bacterial concentration *W*(*t*) and limits the excessive growth when concentration approaches the limiting capacity using the cap parameter *χ*.

The proposed multiplier *h*(*t*) has a novel structure. In the model it is assumed that bacterial growth is linearly related to the current temperature which is controlled by parameter *β*. Precipitation is assumed to have a maximum effect on the bacterial growth at the value *ρ*_*c*_. It is assumed that for smaller amounts of precipitation than *ρ*_*c*_ there is not enough water to wash the bacteria into the environment which causes slower growth. For amounts of precipitation above *ρ*_*c*_ bacteria becomes diluted which diminishes the rate of bacterial growth in the environment. Graphically, the function *h*(*t*) has a bell-shaped curve where *α* and *σ* ^2^ are the calibration parameters of inferential interest.

The last process in the environmental reservoir is the natural decay (death) of bacteria in the environment which is modeled by the time-varying death rate *γ*_*W*−_(*t*).

Please refer to the supplement S1 Text for more technical details on model formulation and assumptions. Overall, the model defined by Eq (1) is neither identifiable (*i.e*., there are too many unknown parameters) nor estimable without extra assumptions [15]. Only precipitation, temperature and the symptomatic compartment *I* (if underreporting is accounted for) can be treated as observed. To summarize, a Susceptible-Infected-Recovered-Susceptible (*SIRS*) model has been implemented, where the *V. cholerae* concentration in the water is modeled via the environmental compartment *W*.

In the model the *SIRS* piece is linked to the reported incidence via the symptomatic compartment *I* using the reporting probability *p*_*r*_. The reported incidence was adjusted before estimation by dividing it by the assumed reporting probability *p*_*r*_. To avoid identifiability issues, extra assumptions about the model parameters and the model itself are made. Since the period of time under consideration was very short, the population size was considered to be constant. Please refer to the supplement S2 Text for more technical details about the model parametrization.

To account for the uncertainty in the deterministic model defined by the ordinary differential Eq (1) stochastic Gaussian terms were introduced into the model equations. The stochastic model was fitted to the reported incidence by using the least squares estimation (LSE) approach. Please refer to the supplement S3 Text for details on stochastic model fitting.

Data were collected from multiple sources. The reported cholera incidence for the Ouest Department of Haiti, including the capital Port-au-Prince, was collected by the Haitian Ministry of Health (Ministère de la Santé Publique et de la Population (MSPP) in French) and compiled by the Pan American Health Organization (PAHO) [16] [4]. The weekly incidence of cholera cases was available from October 17, 2010 until April 27, 2014. Daily precipitation (in millimeters) was obtained from the Tropical Rainfall Measuring Mission (TRMM) satellite data [17], and daily temperatures (in Celsius) were obtained from the Port-au-Prince airport (IATA: PAP) monitoring station. The temperature readings were missing for 14.6% of the dates and the missing values were linearly interpolated. Precipitation data did not have any missingness. The environmental data were aggregated weekly so that it could be aligned with the incidence data. The average weekly temperature *τ*(*t*) and cumulative weekly precipitation *ρ*(*t*) were used as covariates.

**Fig 2:**
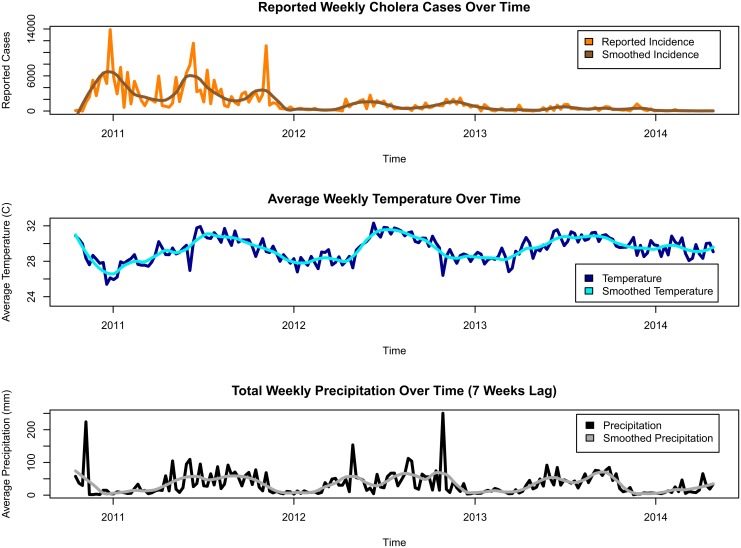
Data collected from the Ouest Department of Haiti. From the top to the bottom: new cases reported weekly, average weekly temperature, and total weekly precipitation with a 7 week lag. Polynomial smoothers (loess function in R) are plotted over each time series to provide better visualization of the mean trends.

In the analysis, it was assumed that there was no time lag for temperature, whereas there was a 7-week lag for precipitation when we evaluated the environmental effects on the water compartment in the model. We did not observe any lag for the temperature from the data. Temperature had only a mild correlation with reported cholera incidence, and the empirical evidence suggested that the association between water temperature and isolations of toxigenic *V. cholerae* from the environment was the strongest with a time lag of 0 to 1 month [6]. At the same time a seven-week lag maximizes the sample correlation between total weekly precipitation and weekly reported cholera incidence. Moreover, there is empirical evidence that the bacteria concentration peaks in the environments three to four weeks after the rainfall, which is associated with an increase in the incidence approximately four weeks later [6]. Thus, a seven week time lag for precipitation was considered plausible. A visual presentation of aligned time series of incidence, temperature and seven-week-lagged precipitation is shown in Fig 2.

The transmissibility of a pathogen in a susceptible population is often measured using the basic reproductive number. Unfortunately, because of the complexity of the model, time-dependent covariates, and the multiple types of sources of infection (humans and the aquatic environment), there was no straightforward epidemiological interpretation of $R_{0}$ for this model. Moreover, in this model $R_{0}$ was technically time dependent because of the time dependent environmental covariates and phage dependent bacterial death rate. The details on the computation of the the basic reproductive number are provided in the supplement S4 Text.

## Results

**Fig 3:**
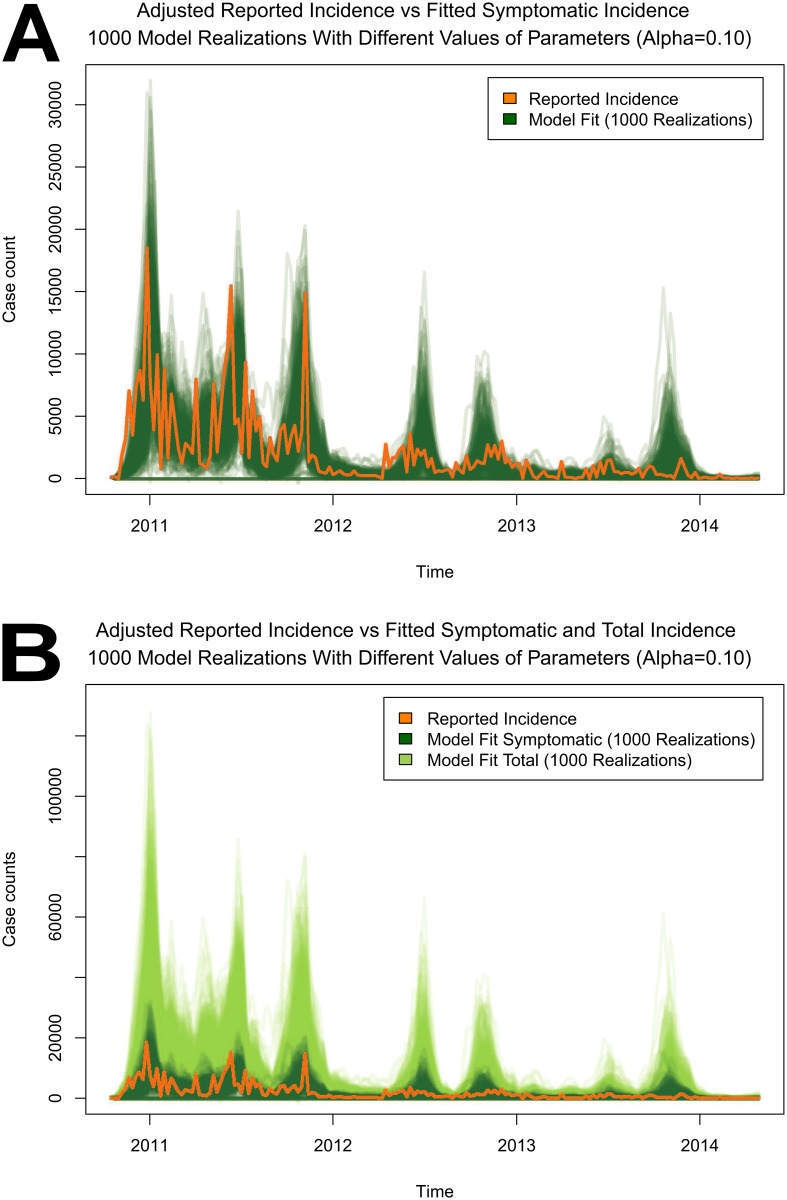
Visual representation of the model fit. A) 1000 Realizations of symptomatic infections produced by the model. B) 1000 Realizations of symptomatic and total infections produced by the model.

The obtained model fit provided a good understanding of the dynamics of the epidemic over time. The visual summary of the model fit together with the adjusted reported cholera incidence is shown in Fig 3. First, the reported incidence was adjusted by rescaling to account for disease underreporting and plotted in orange in Fig 3 for better visual comparison with the model output. To produce the model realizations a different Gaussian white noise time series was generated for each set of 1000 parameter estimates obtained from the previous LSE fits. The corresponding model outputs are displayed in Fig 3. The transparency was tuned to display the density of the curves in each part of the graph and to improve visualization. The symptomatic cases produced by the model are displayed in dark green in panel **A** of Fig 3. The realizations of both the symptomatic (dark green) and total (light green) cases produced by the model are displayed for comparison in panel **B** of Fig 3 on a different scale. The total underlying realizations of the model that include both symptomatic and asymptomatic infections are much larger than the symptomatic realizations alone.

The peak precipitation effect was estimated at ${\hat{\rho }}_{c}=45.1\text{mm}$ with 95% CI (43.0; 47.3) and the threshold parameter for the effect of shedding at $\hat{\delta }=27.0\;\text{mm}$ with 95% CI (6.7; 101.3), which was estimated to be more variable than ${\hat{\rho }}_{c}$. Those estimates did not change much from the starting points that were used for the iterative LSE minimization procedure, indicating the potential lack of information in the data about *ρ*_*c*_ and *σ*. The estimate for the effect of temperature had a median value much higher than the mean value, which indicated a heavy left tail. The median was used over 1000 realizations instead of the mean *β* to provide a more robust estimate. The estimate for $\hat{\beta }$ was 0.014 with 95% CI (−0.041; 0.027), which led to the conclusion that temperature had a mild association with *V. cholerae* growth. The complete list of parameters is provided in Table A in S3 Text.

**Fig 4:**
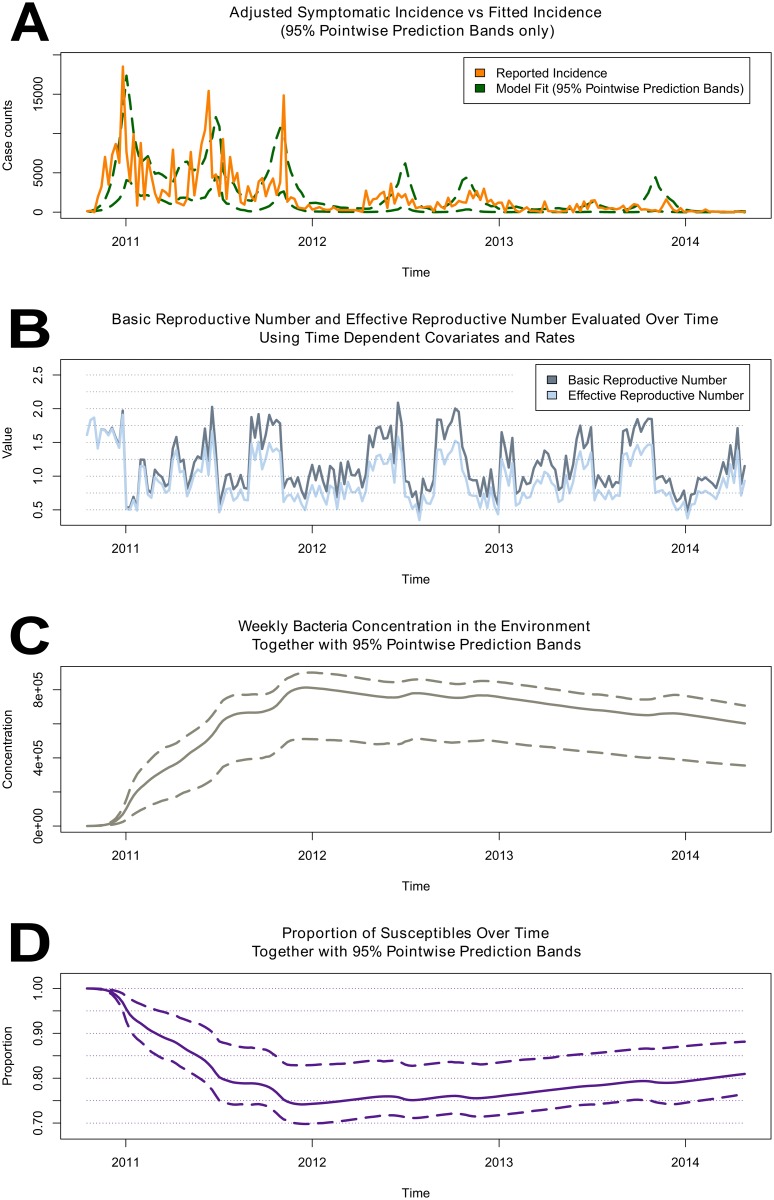
The behavior of the epidemic over time. A) Pointwise prediction bands for the symptomatic infections produced by the model. B) The change in the value of the estimated basic reproductive number ${\hat{R}}_{0}\left(t\right)$ (using extended definition) and in the value of the estimated effective reproductive number $\hat{R}\left(t\right)$ over time. C) *V. cholerae* concentration in the environment over time and the corresponding prediction bands. D) Change in the proportion of the susceptible individuals during the course of the epidemic produced by the model and the corresponding prediction bands.

If a single estimate for $R_{0}$ that summarizes the epidemic behavior is desired, a reasonable approach is to use the averaged values of the time dependent covariates and bacterial death rate to obtain the average estimate ${\hat{R}}_{0}=1.6$ with 95% CI (1.3, 2.1) based on the mean of 1000 stochastic realizations. Alternatively, one may extend the definition of the basic reproductive number to allow for time-dependent covariates and denote it by $R_{0}\left(t\right)$. Readers please refer to the supplement S4 Text for details. Another useful measure is the time-dependent effective reproductive number R(t) which is defined as a product of the basic reproductive number $R_{0}\left(t\right)$ and the proportion of susceptibles at a given time *t*. The change in the value of the estimated basic reproductive number ${\hat{R}}_{0}\left(t\right)$ (using the extended definition) and the estimated effective reproductive number $\hat{R}\left(t\right)$ over time are shown in panel **B** of Fig 4.

Additional characteristics of the epidemic are illustrated in Fig 4. In panel **A** the reported incidence adjusted for underreporting and the pointwise prediction bands for symptomatic infections are displayed. As shown the pointwise prediction bands were able to capture the majority the dynamics of the cholera epidemic. In panel **C** the concentration of *V. cholerae* in the environment and the corresponding prediction bands are shown over time. In panel **D** the changes in the proportion of the susceptible individual during the course of the epidemic produced by the model and corresponding prediction bands are shown.

Based on the trend produced by the model and the observed incidence displayed in panel **A**, it was concluded that the epidemic of cholera likely stabilized in Ouest Department of Haiti after three years of transmission and became endemic. In the model output displayed in panel **D** the proportion of susceptible individuals at the end of the epidemic remains very high and is gradually increasing, which provides the necessary conditions to facilitate further cholera transmission. Furthermore, as displayed in panel **C**, since the concentration of toxigenic *V. cholerae* in the environment produced by the model remains sufficiently high at the end of the observation period, it is also likely that future cholera outbreaks will occur.

## Discussion

In this work, a dynamic model that incorporated the available environmental data was used to describe the transmission of cholera in Ouest Department of Haiti. The model output suggested the existence of a large environmental reservoir of toxigenic *V. cholerae* that reached a peak concentration early in 2012, with a subsequent slow decline (Fig 4). The presence of such an environmental reservoir was consistent with environmental studies conducted in the Leogane flood basin of the Ouest Department, which identified *V. cholerae* O1 in multiple river and estuarine ecosystems [5] [6]. A similar trend was observed in the human susceptible compartment of the model, where the smallest number of susceptible population members was observed in early 2012, with a slow but steady increase since that time (Fig 4).

The model of the cholera epidemic in Haiti described by this study was novel in the way in which the environmental compartment was considered. As previously mentioned, most previous dynamic models of the cholera epidemic in Haiti postulated that toxigenic *V. cholerae* only occupy a transient state in the environment, where pathogenic bacteria shed into the surface water by humans decay at a constant rate and cannot increase without additional cases. This assumption precludes the ability for toxigenic *V. cholerae* to become more prevalent in the environment during periods of decreased cholera incidence and does not explain the resurgence of cholera cases after inter-epidemic periods; both of which have recently been observed in Haiti [4] [6].

As with any mathematical model of infectious disease transmission, this approach was not without limitations. One important theoretical concern was the assumption of homogenous mixing. The contact transition rates between compartments assume homogenous mixing and do not account for the local population density, presence of human mobility networks, and personal hygiene practices within households [18]. Likewise, the contact rates between humans and the environment are also dependent on the proportion of the population that consume contaminated surface water, which varies between urban and rural areas and by demographic factors [19] [20]. Besides the reliance of our model on previously published estimates of some parameters, there are also unobserved processes that occurred during the epidemic, such as increased consumption of bottled water in urban areas of up to 38% and the fluctuation in the number of cholera treatment centers (CTC) as the incidence began to decline [21] [22]. However, the demographic data as well as the number of interventions applied from the international network of aid organizations are also difficult to quantify, making their inclusion in the model speculative at best.

Thus far, only a single serological study of cholera in Haiti was conducted in high-risk populations near the Artibonite River six months after the onset of the epidemic, which reported that 39% of the participants had antibody titers consistent with a recent cholera infection [23]. Our model, which used incidence data from the neighboring Ouest Department, where the onset of the epidemic occurred later, showed a projected population proportion of susceptibles that was somewhat higher at that time. Aside from the one study cited, there have been no further serologic studies reported in Haiti, so it is not possible to comment directly on the validity of the model’s projections. Nonetheless, a rising proportion of susceptibles is plausible, given the anticipated waning of immunity to El Tor cholera over time, and a birth rate that is over 40% higher than other developing countries in Latin America and the Caribbean [24] [25]. The combination of environmental reservoirs of toxigenic *V. cholerae*, lack of adequate sanitation and hygiene infrastructure, and a slowly rising proportion of susceptible population members suggests that seasonal epidemics are likely to be observed in the future. Furthermore, there remains the possibility of major cholera epidemics following hurricanes that generate severe flooding or other environmental disasters that could damage the existing sanitation and drinking water infrastructure. Given the potential for future cholera outbreaks and the demonstrated efficacy of the oral cholera vaccine in Haiti, it would be useful to have epidemic mitigation plans in place that include provisions for the use of the WHO mobile stockpile of cholera vaccine [26] [27].

## Supporting Information

S1 Text Model formulation. This section describes how the proposed model is formulated in terms of ordinary differential equations (ODE), and how the environmental data are incorporated into the model.
(PDF)

S2 Text Model parametrization. Model parameters are defined and explained in this section, including which parameters are fixed at empirical values based on literature review, and which are estimated from the data.
(PDF)

S3 Text Stochastic LSE Approach. How stochastic components are added to the differential equations and how the model is fitted are described in this section.
(PDF)

S4 TextEstimation of the basic reproductive number $R_{0}$.This section gives the definition and derivation of the basic reproductive number $R_{0}$ for the proposed model. The challenges of defining $R_{0}$ in the presence of time-varying transition rates are discussed.(PDF)
