# Correction: Cholera Transmission in Ouest Department of Haiti: Dynamic Modeling and the Future of the Epidemic

**DOI:** 10.1371/journal.pntd.0004377

**Published:** 2016-01-06

**Authors:** Alexander Kirpich, Thomas A. Weppelmann, Yang Yang, Afsar Ali, J. Glenn Morris, Ira M. Longini

In the Funding section, the second grant number from the funder NIH is listed incorrectly. The correct grant number is: U54 GM111274.
