# Supplementary material for: Cholera Transmission in Ouest Department of Haiti: Dynamic Modeling and the Future of the Epidemic
Source: PLoS Negl Trop Dis. 2015 Oct 21;9(10):e0004153. doi: 10.1371/journal.pntd.0004153 (PMC4619523; doi:10.1371/journal.pntd.0004153)
Supplement: S1 Text — This section describes how the proposed model is formulated in terms of ordinary differential equations (ODE), and how the environmental data are incorporated into the model. (PDF) [file pntd.0004153.s001.pdf]

S1 Model Formulation

To bridge the concentration of *V. cholerae* in the environment (W) and the transmission dynamics in humans (S, I, A and R), we adopt a well-accepted logistic dose-response curve

$$f(t) = \frac{W(t)}{\kappa + W(t)}, \tag{2}$$

where $W(t)$ is the concentration of *V. cholerae* in water (cells/ml) and κ is the concentration of *V. cholerae* in water that yields 50% chance of becoming infected with *V. cholerae* (cells/ml) [1] [2] [3] [4]. This logistic dose-response function is biologically plausible and mathematically simple. The estimate of the threshold parameter κ is obtained from the literature.

A novel approach to modeling $W(t)$ by integrating three processes that represent the understanding of the dynamics of toxigenic *V. cholerae* in the environment was proposed.

The first process is the influx of bacteria via shedding by infected human hosts into the environment. The baseline shedding rates are μ_{AW} and μ_{IW} for asymptomatic and symptomatic infections. As flooding moves the bacteria from sewage systems into the aquatic environment, we assumed the effective level of shedding was mediated by precipitation through a logistic dose-response curve $g(t) = \frac{\rho(t)}{\delta+\rho(t)}$ such that the effective rate of bacteria influx was $g(t)(\mu_{AW} + \mu_{IW})$. The parameter δ was interpreted as the threshold parameter corresponding to the half of the maximal possible effect of precipitation. The precipitation level was measured in millimeters per week.

The second process is the multiplication of the bacteria in the environment, which is affected by both precipitation and temperature. An appropriate amount of water may help gather, while too much flooding may wash away nutrients necessary for bacterial growth. The growth of *V. cholerae* has been demonstrated to prefer warmer temperature, particularly above 20°C. We assumed the effect of precipitation on multiplication takes the form of a bell-shaped curve with an exponential decay at the two sides of the peak, similar to the density function of the normal distribution, and the effect of temperature was linear. Their joint effect can be presented as

$$h(t) = \alpha \exp\left[-\frac{(\rho(t) - \rho_c)^2}{2\sigma^2}\right] + \beta\tau(t), \tag{3}$$

where $\tau(t)$ is the temperature at time t, and α and σ, and are the unknown parameters defining the curve. To prevent uncontrolled growth of the bacteria in the environment, we used the logistic population growth function model adjusted for precipitation and temperature, $\frac{\chi-W(t)}{\chi}W(t)$, for the multiplication of the bacteria, where χ is the upper bound of the bacteria concentration in the environment W. Let $m(t) = \frac{\chi-W(t)}{\chi}$. The effective multiplication process is represented by $h(t)m(t)W(t)$.

The last process in the environmental reservoir is the natural decay (death) of bacteria represented by the rate function $\gamma_{W-}(t)$. How to model he bacteria death rate $\gamma_{W-}(t)$ in the environment is an open research question. The simplest approach is to assume a constant rate $\gamma_{W-}(t) = \gamma_{W-}$. However, our preliminary estimation of the parameters for the model indicated that the goodness of fit is poor under such an assumption. Multiple studies have found abrupt decrease in cholera presence following the bloom of toxigenic *V. cholerae* in the environment, and it was hypothesized that

such reduction was a consequence of the invasion of the microorganism by a lytic bacteriophage [5]. Previous research from Bangladesh has also demonstrated seasonal variations in the concentration of the phage in the environment, which was directly related to cholera incidence and indirectly related to the isolations of toxigenic *V. cholerae* from the environment [5] [6].

Combining all the model components, we reach the following deterministic system of ordinary differential equations (ODE):

$$
\begin{aligned}
\frac{dS(t)}{dt} &= \mu_{RS}R(t) - (\mu_{SA}^{W} + \mu_{SI}^{W})S(t)f(t) - (\mu_{SA}^{H} + \mu_{SI}^{H})S(t)\Big(A(t) + I(t)\Big) \\
\frac{dA(t)}{dt} &= \mu_{SA}^{W}S(t)f(t) + \mu_{SA}^{H}S(t)\Big(A(t) + I(t)\Big) - \mu_{AR}A(t) \\
\frac{dI(t)}{dt} &= \mu_{SI}^{W}S(t)f(t) + \mu_{SI}^{H}S(t)\Big(A(t) + I(t)\Big) - \mu_{IR}I(t) \\
\frac{dR(t)}{dt} &= \mu_{AR}A(t) + \mu_{IR}I(t) - \mu_{RS}R(t) \\
\frac{dW(t)}{dt} &= g(t)\Big(\mu_{AW}A(t) + \mu_{IW}I(t)\Big) + h(t)m(t)W(t) - \gamma_{W-}(t)W(t)
\end{aligned}
\tag{4}
$$

References

1. Codeco CT (2001) Endemic and epidemic dynamics of cholera: The role of the aquatic reservoir. BMJ Infect Dis 1:1

2. Hartley DM, Morris JG Jr, Smith DL (2006) Hyperinfectivity: A critical element in the ability of V. cholerae to cause epidemics? PLoS Med 3:7.

3. Mukandavirea Z, Liaob S, Wangc J, Gaffd H, Smitha DL, Morris JG Jr (2011) Estimating the reproductive numbers for the 2008-2009 cholera outbreaks in Zimbabwe. PNAS, 8767-8772, doi: 10.1073/pnas.1019712108.

4. Mukandavire Z, Smith DL, Morris JG Jr (2013) Cholera in Haiti: Reproductive numbers and vaccination coverage estimates. Sci Rep. 3: 997. doi: 10.1038/srep00997.

5. Faruque SM, Islam MJ, Ahmad QS, Faruque AS, Sack DA, Nair GB, and Mekalanos JJ (2005) Self-limiting nature of seasonal cholera epidemics: Role of host-mediated amplification of phage. PNAS Apr 26;102(17):6119-24.

6. Faruque SM, Naser IB, Islam MJ, Faruque AS, Ghosh AN, Nair GB, Sack DA and Mekalanos JJ (2005) Seasonal epidemics of cholera inversely correlate with the prevalence of environmental cholera phages. PNAS Feb 1;102(5):1702-7.
