# Supplementary material for: Cholera Transmission in Ouest Department of Haiti: Dynamic Modeling and the Future of the Epidemic
Source: PLoS Negl Trop Dis. 2015 Oct 21;9(10):e0004153. doi: 10.1371/journal.pntd.0004153 (PMC4619523; doi:10.1371/journal.pntd.0004153)
Supplement: S2 Text — Model parameters are defined and explained in this section, including which parameters are fixed at empirical values based on literature review, and which are estimated from the data. (PDF) [file pntd.0004153.s002.pdf]

S2 Model Parametrization

Since the dimension of parameter space is large, the initial values of some parameters were set to empirical estimates from the literature and confine the search space for these parameters, so that the other key parameters are identifiable. To keep the model simple and identifiable, the population size in Ouest Department was assume to be static, *i.e.*,

$$S(t) + A(t) + I(t) + R(t) = N. \tag{5}$$

This assumption was reasonable since the analysis covered a relatively short period of three years. This assumption could have been relaxed if the birth, death and migration rates in this region during this period were known.

The following is a list of the parameters used in the model.

- $N = 3 \times 10^6$: the population size in the Ouest Department [1].
- $I_0 = I(0)$ and $A_0 = A(0)$: the numbers of symptomatic and asymptomatic infected persons at the beginning of the epidemic ($t = 0$).
- μ_{RS}: the rate of waning of immunity. The rate differs between symptomatic and asymptomatic infections. Immunity lasts from 12 weeks to 3 years [2] [3].
- p_r: the probability that a symptomatic case was reported to the surveillance system. We assume this probability is 0.75 [4].
- μ_{SA}^W or μ_{SI}^W: the rates at which a susceptible person is infected and becomes asymptomatic or symptomatic when exposed to the aquatic environment with unit concentration of toxigenic *V. cholerae*. We assume $\mu_{SA}^W = 3\mu_{SI}^W$ [4]. These two parameters are to be estimated.
- μ_{SA}^H or μ_{SI}^H: the rates at which a susceptible person is infected by an infectious person and becomes asymptomatic or symptomatic. We assume $\mu_{SA}^H = 3\mu_{SI}^H$. These two parameters are to be estimated.
- μ_{IR} or μ_{AR}: the recovery rates of a symptomatic or asymptomatic infection. We assumed $\mu_{IR} = \mu_{AR} = 1$, *i.e.*, an infected recovers on average after one week, regardless of symptom status, based on the range of 3 to 14 days in the literature [3] [5] [6].
- μ_{IW} or μ_{AW}: the bacterial shedding rates from symptomatic or asymptomatic infected individual into the aquatic environment. The shedding rate of an asymptomatic infection may range $0.01 \sim 10$ per day, or equivalently $0.07 \sim 70$ per week [7]. We assume $\mu_{AW} = 0.07$ and $\mu_{IW} = 7$, *i.e.*, the shedding rate of a symptomatic case is 100 times that of an asymptomatic infection.
- κ: the concentration of toxigenic *V. cholerae* (cells/ml) in water that yields 50% chance of becoming infected with cholera (cells/ml) [8]. Its value ranges from 10^5 to 10^6 depending on the conditions of the water [7]. A sensitivity analysis was performed for the values in this range to improve the model performance and at the same time to preserve biological plausibility. The value $\kappa = 10^5$ was used in the final analysis.

- W: the concentration of toxigenic *V. cholerae* in the aquatic environment. We assume it is bounded below by 1 ml^{-1}, which is an arbitrary level we consider as sufficiently low, and is bounded above by $\chi = 10^6$ [9]. The initial level at time $t = 0$ is assumed to be $10^2\,\mathrm{ml}^{-1}$ [9].

- δ: the parameter is interpreted as the threshold corresponding to half of the maximal possible effect of precipitation. A reasonable range was found to be $15 - 45$ mm [10]. We assumed $\delta = 30$ mm as a starting point for the estimation procedure.

- α and β: the coefficients of precipitation and temperature for the multiplication process of the bacteria in the aquatic environment. Their values are to be estimated.

- ρ_c: the precipitation value necessary to reach the peak level of the bacterial growth rate in the aquatic environment. We assume $\rho_c = 45$ mm according to [10] as a starting point for the estimation procedure.

- σ: the scaling factor for the effect of precipitation on the multiplication process of the bacteria in the aquatic environment. Based on the assumed values of δ and ρ_c, we use $\sigma = 15$ as a starting point for the estimation procedure to obtain a bell-shaped curve resembling the shape of the normal density function.

- r: another shape parameter for the functional form of the effect of precipitation on the multiplication process of the bacteria in the aquatic environment. We assumed that for small precipitation values the effect is negligible. For certain peak values of precipitation we see the maximum effect on bacterial multiplication and for the higher values of precipitation the bacteria becomes getting diluted and the effect of precipitation decreases. This relationship is modeled via bell-shaped curve with $r = 2$.

- γ_{W-}: bacteria death rate, which is not well investigated. The bacteria can live in the environment from 3 to 41 days, or equivalently 0.42-5.86 weeks, depending on the water characteristics [7]. We set the initial value of γ_{W-} to be 0.36, corresponding to a life expectancy of 2.8 weeks [7]. This parameter is to be estimated. We multiply γ_{W-} by a time-varying weight ranging between 0 and 1 to reflect the impact of phage. The exact functional form of this phage effect is tuned to reach a reasonable fit of the model to the data. [11] Please refer to Fig. A for more details on the weights.

- Based on the cross-correlation estimates, no lag was utilized for the temperature and a 7 week lag was used for the precipitation. The cross-correlation function for the reported incidence and precipitation is presented in Fig. B.

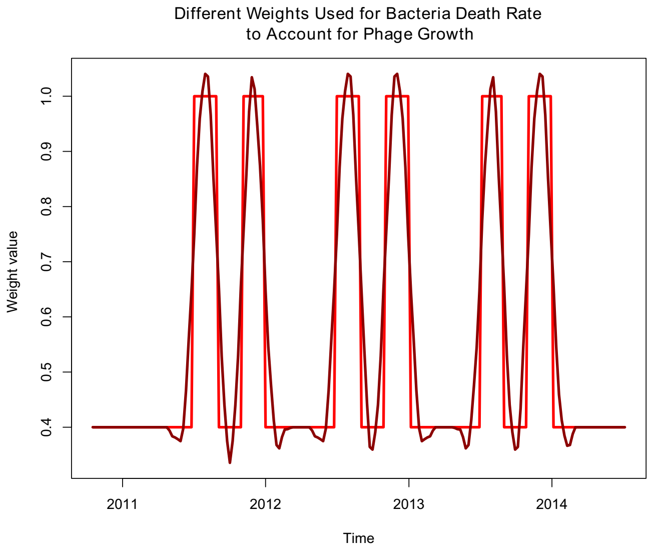

Figure A. Weights Used for Scaling Bacteria Death Rate. The piecewise-constant weights were used in the final analysis. Smoothed weights using polynomial smoothers (`loess` function in `R`) yielded similar results.

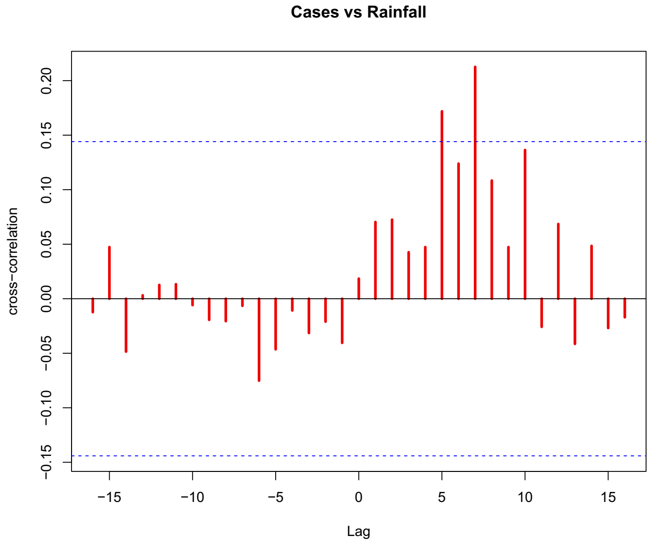

Figure B. Cross-correlation of the reported cases and precipitation levels. The cross-correlation of the reported cases and precipitation is shown. A seven weeks lag was chosen for the analysis and model fitting.

References

1. World Public Library (2014) Departments of Haiti. http://www.worldlibrary.org/articles/Departments_of_Haiti Accessed May 2015.

2. Koelle K, Rodo X, Pascual M, Yunus M, Mustafa G (2005) Refractory periods and climate forcing in cholera dynamics. Nature. 436:696-700.

3. Righetto L, Bertuzzo E, Mari L, Schild E, Casagrandi R, Gatto M, Rodriguez-Iturbe I, Rinaldo A (2013) Rainfall mediations in the spreading of epidemic cholera. Advances in Water Resources.

4. Bertuzzo E, Finger F, Mari L, Gatto M, Rinaldo A (2014) On the probability of extinction of the Haiti cholera epidemic. Stochastic Environmental Research and Risk Assessment.

5. Kaper JB, Morris JG Jr, Levine MM (1995) Cholera. Clin Microbiol Rev. Jan;8(1):48-86.

6. Nelson EJ, Harris JB, Calderwood SB, Camilli A (2009) Cholera transmission: the host, pathogen and bacteriophage dynamic. Nat Rev Microbiol 7:693-702.

7. Fung IH (2014) Cholera transmission dynamic models for public health practitioners. Emerging Themes in Epidemiology 11(1):1-11.

8. Codeco CT (2001) Endemic and epidemic dynamics of cholera: The role of the aquatic reservoir. BMJ Infect Dis 1:1

9. Vital M, Fuchslin HP, Hammes F, Egli T (2007) Growth of Vibrio cholerae O1 Ogawa Eltor in freshwater. Microbiology.

10. Hashizume M, Armstrong B, Hajat S, Wagatsuma Y, Faruque AS, Hayashi T, Sack DA (2008) The effect of rainfall on the incidence of cholera in Bangladesh. Epidemiology. Jan;19(1):103-10.

11. Faruque SM, Islam MJ, Ahmad QS, Faruque AS, Sack DA, Nair GB, and Mekalanos JJ (2005) Self-limiting nature of seasonal cholera epidemics: Role of host-mediated amplification of phage. PNAS Apr 26;102(17):6119-24.
