# Supplementary material for: Cholera Transmission in Ouest Department of Haiti: Dynamic Modeling and the Future of the Epidemic
Source: PLoS Negl Trop Dis. 2015 Oct 21;9(10):e0004153. doi: 10.1371/journal.pntd.0004153 (PMC4619523; doi:10.1371/journal.pntd.0004153)
Supplement: S3 Text — How stochastic components are added to the differential equations and how the model is fitted are described in this section. (PDF) [file pntd.0004153.s003.pdf]

S3 Stochastic LSE Approach

To introduce stochasticity into the model, we follow the approach in [1] by adding stochastic components to the differential equations (4):

$$
\begin{aligned}
\frac{dS(t)}{dt} =& \mu_{RS}R(t) - (\mu_{SA}^{W} + \mu_{SI}^{W}) \Big[1 + \varphi_W \xi^W(t)\Big] S(t)f(t) - \\
& (\mu_{SA}^{H} + \mu_{SI}^{H}) \Big[1 + \varphi_H \xi^H(t)\Big] S(t)\Big(A(t) + I(t)\Big) \\
\frac{dA(t)}{dt} =& \mu_{SA}^{W} \Big[1 + \varphi_W \xi^W(t)\Big] S(t)f(t) + \mu_{SA}^{H} \Big[1 + \varphi_H \xi^H(t)\Big] S(t)\Big(A(t) + I(t)\Big) - \mu_{AR}A(t) \\
\frac{dI(t)}{dt} =& \mu_{SI}^{W} \Big[1 + \varphi_W \xi^W(t)\Big] S(t)f(t) + \mu_{SI}^{H} \Big[1 + \varphi_H \xi^H(t)\Big] S(t)\Big(A(t) + I(t)\Big) - \mu_{IR}I(t) \\
\frac{dR(t)}{dt} =& \mu_{AR}A(t) + \mu_{IR}I(t) - \mu_{RS}R(t) \\
\frac{dW(t)}{dt} =& g(t)\Big(\mu_{AW}A(t) + \mu_{IW}I(t)\Big) + h(t)m(t)W(t) - \gamma_{W-}(t)W(t),
\end{aligned}
\tag{6}
$$

where $\xi^W(t)$ and $\xi^H(t)$ are two independent Gaussian white noise processes. Here φ_W and φ_H are standard deviations (to be estimated) that characterize the uncertainty in the dynamics.

The parameters of interest to be estimated from this stochastic system are

$$
\boldsymbol{\theta} = (\mu_{SI}^{W}, \mu_{SI}^{H}, \mu_{RS}, \gamma_{W-}, \alpha, \beta, \rho_c, \sigma, \delta, \varphi_W, \varphi_H). \tag{7}
$$

The estimation procedure proceeds as follows [1]:

for $m = 1, \ldots, M$ **do**

Generate independent noise time series $\xi^W(t)_m$ and $\xi^H(t)_m$ at points $t = 1, \ldots, T$.

Solve the differential equations with the stochastic terms specified by (6) numerically for the generated $\xi^W(t)_m$ and $\xi^H(t)_m$.

Use the least squares approach to find the values of the parameters that minimize the sum of squares and record the values of the parameters in the vector $\boldsymbol{\theta}_m$.

end for

At each iteration we fit the model using the `optim` function in `R` to minimize the sum of squared differences between data and the model. We repeat the process of generating white noise time series and fitting the model for $M = 1000$ times. As a result for each parameter we had 1000 estimates. To produce the 95% confidence intervals we ordered the estimates for each parameter and picked 2.5% percentile and 97.5% percentile (25th and 976th sorted estimates to be precise.) The estimates and the confidence intervals produced in this way do not rely on normality or any other assumption. The estimates for parameters of interest and confidence intervals are summarized in Table A in S3 Stochastic LSE Approach. The postulated parameters are summarized in Table B in S3 Stochastic LSE Approach.

In case non-logarithmic scale values for the parameters listed in Table A in S3 Stochastic LSE Approach are desired, the exponentiated values are provided in Table C in S3 Stochastic LSE Approach.

Table A. Summary of the parameter estimates for the 2010-2014 cholera epidemic in Haiti. Estimates are based on the model with stochastic components and are obtained from 1000 sets of randomly generated white noise time series. Nonnegative parameters are provided on the logarithmic scale.

Parameter	Smallest	Low 95%	Median	Mean	High 95%	Largest
$\log(\mu_{SI}^W)$	-4.849	-4.694	-4.290	-4.276	-3.849	-3.849
$\log(\mu_{SA}^W)^\dagger$	-3.750	-3.595	-3.191	-3.178	-2.750	-2.750
$\log(\mu_{SI}^H)$	-25.253	-25.051	-25.001	-24.999	-24.933	-24.501
$\log(\mu_{SA}^H)^\ddagger$	-24.155	-23.952	-23.902	-23.834	-23.864	-24.006
$\log(\mu_{RS})$	-5.318	-5.318	-5.282	-5.227	-5.025	-4.282
$\log(\gamma_{W-})$	0.402	0.515	0.866	0.859	1.250	1.946
$\log(\alpha)$	-20.376	-20.045	-19.999	-19.999	-19.974	-19.726
β	-4.980	-0.041	0.014	-0.049	0.027	0.041
$\log(\rho_c)$	3.649	3.761	3.806	3.808	3.856	4.385
$\log(\sigma)$	2.447	2.654	2.707	2.705	2.742	2.944
$\log(\delta)$	1.521	1.907	3.663	3.292	4.618	8.701
$\log(\varphi_W)$	-4.493	-4.493	-2.110	-2.378	-0.694	-0.688
$\log(\varphi_H)$	2.066	2.717	2.773	2.773	2.829	2.996

†: $\log(\mu_{SA}^W) = \log(3\mu_{SI}^W)$

‡: $\log(\mu_{SA}^H) = \log(3\mu_{SI}^H)$

Table B. Parameters that are fixed when fitting the model with stochastic components to the 2010-2014 cholera epidemics in Haiti.

Parameter	μ_{AR}	μ_{IR}	μ_{AW}	$\mu_{IW} = 100\mu_{AW}$	κ	χ
Value	1	1	0.07	7	10^5	10^6

Table C. Summary of the parameter estimates for the 2010-2014 cholera epidemic in Haiti. Estimates are based on the model with stochastic components and are obtained from 1000 sets of randomly generated white noise time series. Nonnegative parameters are provided on the normal scale without logarithms.

Parameter	Smallest	Low 95%	Median	Mean	High 95%	Largest
μ_{SI}^W	0.008	0.009	0.014	0.014	0.021	0.021
$\mu_{SA}^{W\dagger}$	0.024	0.027	0.041	0.042	0.064	0.064
μ_{SI}^H	1.078e-11	1.320e-11	1.387e-11	1.390e-11	1.485e-11	2.287e-11
$\mu_{SA}^{H\ddagger}$	3.233e-11	3.961e-11	4.164e-11	4.457e-11	4.325e-11	1.020e-10
μ_{RS}	0.005	0.005	0.005	0.005	0.007	0.014
γ_{W-}	1.495	1.674	2.377	2.361	3.490	7.001
α	1.415e-09	1.970e-09	2.063e-09	2.063e-09	2.115e-09	2.711e-09
β	-4.980	-0.041	0.014	-0.049	0.027	0.041
ρ_c	38.436	42.991	44.970	45.060	47.276	80.238
σ	11.554	14.211	14.984	14.954	15.518	18.992
δ	4.577	6.733	38.978	26.897	101.291	6008.918
φ_W	0.011	0.011	0.121	0.093	0.500	0.502
φ_H	7.893	15.135	16.006	16.006	16.928	20.005

†: $\mu_{SA}^W = 3\mu_{SI}^W$

‡: $\mu_{SA}^H = 3\mu_{SI}^H$

References

1. Mukandavire Z, Smith DL, Morris JG Jr (2013) Cholera in Haiti: Reproductive numbers and vaccination coverage estimates. Sci Rep. 3: 997.
