# Supplementary material for: Cholera Transmission in Ouest Department of Haiti: Dynamic Modeling and the Future of the Epidemic
Source: PLoS Negl Trop Dis. 2015 Oct 21;9(10):e0004153. doi: 10.1371/journal.pntd.0004153 (PMC4619523; doi:10.1371/journal.pntd.0004153)
Supplement: S4 Text — This section gives the definition and derivation of the basic reproductive number R0 for the proposed model. The challenges of defining R0 in the presence of time-varying transition rates are discussed. (PDF) [file pntd.0004153.s004.pdf]

S4 Computation of $\mathcal{R}_0$

The basic reproductive number, $\mathcal{R}_0$, is usually defined as the average number of new infections an infected person can generate during the infectious period when introduced in the totally susceptible population, *i.e.*, at the point of disease free equilibrium (DFE). Following the notation in [1], let $\boldsymbol{X}_t = (A_t, I_t, W_t, S_t, R_t)$, where compartments are re-ordered such that those with inflow of new infections appear first. The simplest DFE point is $\boldsymbol{X}_0 = (0, 0, 0, N, 0)$, but here we provide formula for any arbitrary time point $\boldsymbol{X}_t$. Define the following notation:

- $\mathcal{F}_i(\boldsymbol{X}_t)$: the rate of increase in compartment i due to inflow of new infections.
- $\mathcal{V}_i(\boldsymbol{X}_t)^+$: the rate of increase in compartment i due to reasons other than new infections.
- $\mathcal{V}_i(\boldsymbol{X}_t)^-$: the rate of outflow of compartment i due to any reasons.
- $\mathcal{V}_i(\boldsymbol{X}_t) = \mathcal{V}_i(\boldsymbol{X}_t)^- - \mathcal{V}_i(\boldsymbol{X}_t)^+$: the rate of net outflow unrelated to new infections.

Analogous to the vector-host model [1], we can view the aquatic environment, W, as a vector. For calculating $\mathcal{R}_0$, we only need to focus on the A, S and W compartments, for which we have

$$\boldsymbol{\mathcal{F}}(\boldsymbol{X}_t) = \begin{pmatrix} \mu_{SA}^W S_t f(t) + \mu_{SA}^H S_t (A_t + I_t) \\ \mu_{SI}^W S_t f(t) + \mu_{SI}^H S_t (A_t + I_t) \\ g(t) \left[\mu_{AW} A_t + \mu_{IW} I_t\right] \end{pmatrix} \tag{8}$$

and

$$\boldsymbol{\mathcal{V}}(\boldsymbol{X}_t) = \begin{pmatrix} \mu_{AR} A_t \\ \mu_{IR} I_t \\ \gamma_{W-}(t) W_t - h(t) m(t) W_t \end{pmatrix} \tag{9}$$

where $f(t) = \frac{W_t}{\kappa + W_t}$, $g(t) = \frac{\rho(t)}{\delta + \rho(t)}$, $h(t) = \alpha \exp\left[-\frac{(\rho(t) - \rho_c)^2}{2\sigma^2}\right] + \beta\tau(t)$, and $m(t) = \frac{\chi - W_t}{\chi}$. Let $\boldsymbol{X}_t^\star = (A_t, I_t, W_t)$, and let $\boldsymbol{F}(\boldsymbol{X}_t) = \frac{\partial \boldsymbol{\mathcal{F}}(\boldsymbol{X}_t)}{\partial \boldsymbol{X}_t^\star}$ and $\boldsymbol{V}(\boldsymbol{X}_t) = \frac{\partial \boldsymbol{\mathcal{V}}(\boldsymbol{X}_t)}{\partial \boldsymbol{X}_t^\star}$ be the 3×3 Jacobian matrices.

$$\boldsymbol{F}\boldsymbol{V}^{-1}(\boldsymbol{X}_0) = \begin{pmatrix} \frac{\mu_{SA}^H}{\mu_{AR}} N & \frac{\mu_{SA}^H}{\mu_{IR}} N & \frac{\mu_{SA}^W \frac{N}{\kappa}}{\gamma_{W-}(t) - h(t)} \\ \frac{\mu_{SI}^H}{\mu_{AR}} N & \frac{\mu_{SI}^H}{\mu_{IR}} N & \frac{\mu_{SI}^W \frac{N}{\kappa}}{\gamma_{W-}(t) - h(t)} \\ \frac{\mu_{AW}}{\mu_{AR}} g(t) & \frac{\mu_{IW}}{\mu_{IR}} g(t) & 0 \end{pmatrix} \tag{10}$$

We leave $g(t)$, $h(t)$ and $\gamma_{W-}(t)$ as time-dependent, because they are independent of all the compartments but are dependent on external variables such as precipitation, temperature, and the presence of phage. Assume $\mu_{SA}^H = a\mu_{SI}^H$, $\mu_{SA}^W = a\mu_{SI}^W$, $\mu_{AR} = \mu_{IR}/c$ and $\mu_{AW} = \mu_{IW}/b$, we get

$$\mathcal{R}_0 = \frac{1}{2} \left\{ \sqrt{\frac{4[\frac{ac}{b} + 1]\mu_{SI}^W \frac{N}{\kappa} g(t) \frac{\mu_{IW}}{\mu_{IR}}}{\gamma_{W-}(t) - h(t)} + [ac+1]^2 \left[\frac{\mu_{SI}^H S_t}{\mu_{IR}}\right]^2} + [ac+1] \frac{\mu_{SI}^H S_t}{\mu_{IR}} \right\}.$$

Substituting in $a = 3$ (since asymptomatic infections are assumed to be three times more likely than symptomatic), $b = 100$ (since symptomatic individuals are assumed to

shed a hundred times more *V. cholerae* into the environment than symptomatic individuals), $c = 1$ (since symptomatic and asymptomatic individuals are assumed to have the same average length of infection) we obtained the formula for $\mathcal{R}_0$ evaluation at a given time t.

$\mathcal{R}_0$ is technically time dependent *i.e.* we have $\mathcal{R}_0(t)$ which does not comply with the classical definition of the basic reproductive number for the simplest model which is time independent. This is caused by the complexity of the model that includes time-dependent environmental covariates and the environmental compartment W. If one single basic reproductive number that summarizes the epidemic behavior is desired, the averaged values of the time dependent covariates and rate: $\bar{\rho}$, $\bar{\tau}$ and $\overline{\gamma_{W-}}$ can be used. This substitution led to a single estimate $\hat{\mathcal{R}}_0 = 1.6$ evaluated at point $\boldsymbol{X}_0 = (0, 0, 0, N, 0)$ as an average from 1000 stochastic realizations. The corresponding 95% confidence interval based on 1000 stochastic realizations of the model was (1.3, 2.1).

The extended time dependent definition of the basic reproductive number $\mathcal{R}_0(t)$ could also be used. The value of $\mathcal{R}_0(t)$ at each time point t using the corresponding values of the time dependent rates: $\rho(t)$, $\tau(t)$ and $\gamma_{W-}(t)$ and $\boldsymbol{X}_0 = (0, 0, 0, N, 0)$ were evaluated.

The alternative is to look at the effective reproductive number $\mathcal{R}(t)$, which is by definition time dependent and defined as a product of the basic reproductive number and the proportion of the population that is susceptible at a given time t. The change in the value of the estimated basic reproductive number $\hat{\mathcal{R}}_0(t)$ (using the extended definition) and in the value of the estimated effective reproductive number $\hat{\mathcal{R}}(t)$ over time are provided in panel **B** of Fig. 4.

References

1. Driessche (van den) P, Watmough J (2002) Reproduction numbers and sub-threshold endemic equilibria for compartmental models of disease transmission. Mathematical Biosciences 180 p.29-48
